# Eprobe mediated RT-qPCR for the detection of leukemia-associated fusion genes

**DOI:** 10.1371/journal.pone.0202429

**Published:** 2018-10-03

**Authors:** Koji Tsuchiya, Yoko Tabe, Tomohiko Ai, Takahiro Ohkawa, Kengo Usui, Maiko Yuri, Shigeki Misawa, Soji Morishita, Tomoiku Takaku, Atsushi Kakimoto, Haeun Yang, Hiromichi Matsushita, Takeshi Hanami, Yasunari Yamanaka, Atsushi Okuzawa, Takashi Horii, Yoshihide Hayashizaki, Akimichi Ohsaka

**Affiliations:** 1 Division of Clinical Laboratory, Juntendo University Hospital, Tokyo, Japan; 2 Department of Transfusion Medicine and Stem Cell Regulation, Graduate School of Medicine, Juntendo University, Tokyo, Japan; 3 Department of Next Generation Hematology Laboratory Medicine, Graduate School of Medicine, Juntendo University, Tokyo, Japan; 4 Department of Clinical Laboratory Medicine, Graduate School of Medicine, Juntendo University, Tokyo, Japan; 5 Nucleic Acid Diagnostic System Development Unit, Division of Genomic Technologies, RIKEN Center for Life Science Technologies, Kanagawa, Japan; 6 Genetic Diagnosis Technology Unit, Division of Genomic Technologies, RIKEN Center for Life Science Technologies, Kanagawa, Japan; 7 Department of Hematology, Juntendo University School of Medicine, Tokyo, Japan; 8 Division of Pathology and Clinical Laboratories, National Cancer Center Hospital, Tokyo, Japan; 9 Preventive Medicine and Diagnosis Innovation Program, RIKEN, Wako, Japan; 10 Innovative Medical Technology Research & Development Center, Juntendo University School of Medicine, Tokyo, Japan; European Institute of Oncology, ITALY

## Abstract

The detection and quantification of leukemia-associated fusion gene transcripts play important roles in the diagnosis and follow-up of leukemias. To establish a standardized method without interlaboratory discrepancies, we developed a novel one-step reverse transcription quantitative PCR (RT-qPCR) assay, called “the Eprobe leukemia assay,” for major and minor *BCR-ABL1*, *RUNX1-RUNX1T1*, and various isoforms of *PML-RARA*. This assay is comprised of Eprobes that are exciton-controlled hybridization-sensitive fluorescent oligonucleotides. Melting curve analyses were performed on synthetic quantitative standard RNAs with strict quality control. Quantification capacity was evaluated by comparison with TaqMan RT-qPCR using 67 primary leukemia patient samples. The lower limit of detection and the limit of quantification of this assay were less than 31.3 copies/reaction and 62.5 copies/reaction, respectively. This assay correctly detected the fusion genes in samples with 100% sensitivity and specificity. The specificity of the reactions was confirmed by melting curve analyses. The assay detected low-level expression of minor *BCR-ABL1* co-expressed with major *BCR-ABL1*. These results illustrate the feasibility and high accuracy of the Eprobe leukemia assay, even for minimal residual disease monitoring.

## Introduction

Chromosomal translocation-generated fusion genes in myeloid and lymphoid leukemia are well-known to be associated with prognosis of leukemias.[1–3] Various molecular genetic tests have been developed and used in the treatments of leukemias: (1) to make more accurate diagnoses; (2) to evaluate the risk stratification; (3) to determine target-specific treatments; and (4) to monitor the recurrence such as minimal residual disease.[1–6]

Recently, the College of American Pathologists (CAP) and the American Society of Hematology (ASH) collaborated to develop an evidence-based clinical practice guideline, the "Initial Diagnostic Workup of Acute Leukemia.”[7] In the guideline, it is emphasized that molecular genetic tests should be evaluated in addition to conventional karyotyping based on assessment of the morphologic and immunophenotypic features.[7] For example, rapid detection of *PML-RARA* should be a priority for patients with suspected acute promyelocytic leukemia, detection of *BCR-ABL1* should be examined in adult patients with suspected or confirmed B-cell acute lymphoblastic leukemia, and mutational analyses should be performed in *RUNX1-RUNX1T1*-positive acute leukemia patients.[7] Although molecular tests have been routinely performed in many clinical laboratories following the guideline, the protocols were not standardized for a while.[8–10] Therefore, the World Health Organization (WHO) international standard (IS) have been developed to standardize the detection protocol of the major *BCR-ABL1* fusion gene of chronic myeloid leukemia (CML).[11, 12] Currently, several commercial kits for diagnosis of CML using the WHO-IS are available for detection of the major *BCR-ABL1* (e.g., Armored RNA Quant IS Calibrator Panel (ARQ IS), Asuragen, Inc., Austin, TX, USA). However, these tests using IS and TaqMan probes are too expensive to measure small sized samples, and the method itself is time and labor consuming. To solve these issues, we sought to develop a simpler and dependable single-tube reaction method for the screening and quantitative detection of fusion gene transcripts using PCR-Eprobe melting curve technique, so called “Eprobe mediated RT-qPCR assay.” An Eprobe is a 3’-end-blocked exciton-controlled hybridization-sensitive fluorescent oligonucleotide (ECHO)[13] that contains two dye moieties attached to the same nucleotide. Its fluorescent signal is strongly suppressed as a single-stranded oligonucleotide by an excitonic interaction between the dyes. Upon hybridization to a complementary DNA strand, the dyes are separated and intercalated into the double strand, which generates strong fluorescence signals. The stabilized DNA/DNA hybrid provides an increased melting temperature compared to the standard DNA oligonucleotides when the reaction tubes are heated. Therefore, Eprobes allow for specific real-time monitoring of amplification reactions by hybridizing to the amplicon in a highly specific sequence-dependent manner. Thus, it enables us to analyze reaction products by melting curve analyses[14] which is the main advantage of the Eprobe RT-qPCR assay avoiding gel electrophoresis and sequencing, and contributes to a rapid and an accurate diagnosis. The implementation of synthetic positive control RNAs within the Eprobe RT–qPCR assay provides routine performance control of the tests and guarantees high-quality standards. During the verification, we also overcame off-target amplification by comparing the efficiency of various primer pairs.

## Materials and methods

### Patient samples

A total of 67 RNA samples previously extracted from bone marrow of patients who suffered leukemias were employed.[15] The samples included 31 molecularly positive and 36 molecularly negative for major and minor *BCR-ABL1*, *RUNX1-RUNX1T1*, or *PML-RARA*. This study was reviewed and approved by the Institutional Review Board of Juntendo University. All samples were fully anonymized before we accessed them, and all patients were provided with informed written consent to have their samples/data from their medical records used in research.

### Control RNA isolated from human cell lines

To evaluate the minor *BCR/ABL1* primer sets, we used total RNAs derived from minor and major *BCR/ABL1*-negative HL60 human leukemia cell lines.[16] The HL60 cells were obtained from the American Type Culture Collection (Manassas, VA, USA). The cultured cells (6×10^7^ cells) were homogenized using a Tissue Ruptor (QIAGEN, Venlo, the Netherlands), and total RNA was isolated from homogenized cells using RNeasy Midi Kit (QIAGEN). In each Eprobe RT-qPCR experiment, 1 μg of total RNA was added to the reaction mixture.

### Production of synthetic RNA

Synthetic DNA molecules were designed to contain target sequences for RT-qPCR assays for major and minor *BCR-ABL1*, *RUNX1-RUNX1T1*, *PML-RARA* (bcr1, bcr2, and bcr3 variants), and *cABL* (Table 1). The oligonucleotides were synthesized (Eurofins Genomics, Japan), and cloned into the pTAKN-2 vector (BioDynamics Laboratory, Tokyo, Japan). Linear DNAs were amplified from the plasmids by PCR using a forward primer (GCCAGATCTTAATACGAC) and a reverse primer (CAGGAAACAGCTATGACC). The amplified linear DNAs were purified using NucleoSpin Gel and PCR Clean-up kit (TaKaRa Bio, Inc., Kusatsu, Japan), and *in vitro*-transcribed using the T7 RiboMAX Express Large Scale RNA Production System (Promega, Madison, WI, USA). Concentrations of the RNAs were measured with Nanodrop ND-1000 spectrophotometer (Thermo Fisher Scientific, Wilmington, MA, USA), and then converted to molar units. To evaluate various parameters related to the fusion transcripts, the synthetic RNAs were serially diluted as the following concentrations: 5.2, 10.4, 20.8, 31.3, 62.5, 12.5 × 10^2^, 2.5 × 10^4^, 5.0 × 10^5^, and 1.0 × 10^7^ copies/reaction.

**Table 1:** Sequences of the synthetic DNAs.

*Major BCR-ABL1*	TAATACGACTCACTATAGGGGAAGAAGTGTTTCAGAAGCTTCTCCCTGACATCCGTGGAGCTGCA GATGCTGACCAACTCGTGTGTGAAACTCCAGACTGTCCACAGCATTCCGCTGACCATCAATAAG GAAGATGATGAGTCTCCGGGGCTCTATGGGTTTCTGAATGTCATCGTCCACTCAGCCACTGGATT TAAGCAGAGTTCAAaagcccttcagcggccagtagcatctgactttgagcctcagggtctgagtgaagccgctcgttggaactccaagg aaaaccttctcgctggacccagtgaaatgctagttattgctcagcggccgc
*minor BCR-ABL1*	TAATACGACTCACTATAGGGGTTGTCGTGTCCGAGGCCACCATCGTGGGCGTCCGCAAGACCGG GCAGATCTGGCCCAACGATGGCGAGGGCGCCTTCCATGGAGACGCAGaagcccttcagcggccagtagcatct Gactttgagcctcagggtctgagtgaagccgctcgttggaactccaaggaaaaccttctcgctggacccagtgaaaatgaccccaaccatgctagtt attgctcagcggccgc
*RUNX1-RUNX1T1*	TAATACGACTCACTATAGGGAAATGCTACCGCAGCCATGAAGAACCAGGTTGCAAGATTTAATG ACCTCAGGTTTGTCGGTCGAAGTGGAAGAGGGAAAAGCTTCACTCTGACCATCACTGTCTTCAC AAACCCACCGCAAGTCGCCACCTACCACAGAGCCATCAAAATCACAGTGGATGGGCCCCGAGA ACCTCGAAatcgtactgagaagcactccacaatgccagactcacctgtggatgtgaagacgcaatctaggctgactcctccaacaatgcca Cctcccccaactactcaaggagctccaagaaccagttcatttacaccgacaacgttaactaatggcacgagccattctcctacagccttgaatatg ctagttattgctcagcggccgc
*PML-RARA* (F1)	TAATACGACTCACTATAGGGGCGCTGGTGCAGAGGATGAAGTGCTACGCCTCGGACCAGGAGGT GCTGGACATGCACGGTTTCCTGCGCCAGGCGCTCTGCCGCCTGCGCCAGGAGGAGCCCCAGAGC CTGCAAGCTGCCGTGCGCACCGATGGCTTCGACGAGTTCAAGGTGCGCCTGCAGGACCTCAGCT CTTGCATCACCCAGGGGAAAGATGCAGCTGTATCCAAGAAAGCCAGCCCAGAGGCTGCCAGCAC TCCCAGGGACCCTATTGACGTTGACCTGCCCGAGGAGGCAGAGAGAGTGAAGGCCCAGGTTCAG GCCCTGGGGCTGGCTGAAGCCCAGCCTATGGCTGTGGTACAGTCAGTGCCCGGGGCACACCCCG TGCCAGTGTACGCCTTCTCCATCAAAGGCCCTTCCTATGGAGAGGATGTCTCCAATACAACGACA GCCCAGAAGAGGAAGTGCAGCCAGACCCAGTGCCCCAGGAAGGTCATCAAGATGGAGTCTGAGG AGGGGAAGGAGGCAAGGTTGGCTCGGAGCTCCCCGGAGCAGCCCAGGCCCAGCACCTCCAAGGC AGTCTCACCACCCCACCTGGATGGACCGCCTAGCCCCAGGAGCCCCGTCATAGGAAGTGAGGTCT TCCTGCCCAACAGCAACCACGTGGCCAGTGGCGCCGGGGAGGCAGCCATTGAGACCCAGAGCAG CAGTTCTGAagagatagtgcccagccctccctcgccaccccctctaccccgcatctacaagccttgctttgtctgtcaggacatgctagttattgc tcagcggccgc
*PML-RARA* (F2)	TAATACGACTCACTATAGGGGCGCTGGTGCAGAGGATGAAGTGCTACGCCTCGGACCAGGAGGT GCTGGACATGCACGGTTTCCTGCGCCAGGCGCTCTGCCGCCTGCGCCAGGAGGAGCCCCAGAGC CTGCAAGCTGCCGTGCGCACCGATGGCTTCGACGAGTTCAAGGTGCGCCTGCAGGACCTCAGCT CTTGCATCACCCAGGGGAAAGATGCAGCTGTATCCAAGAAAGCCAGCCCAGAGGCTGCCAGCAC TCCCAGGGACCCTATTGACGTTGACCTGCCATTGAGACCCAGAGCAGCAGTTCTGAagagatagtgccc agccctccctcgccaccccctctaccccgcatctacaagccttgctttgtctgtcaggacatgctagttattgctcagcggccgc
*PML-RARA* (F3)	TAATACGACTCACTATAGGGGCGCTGGTGCAGAGGATGAAGTGCTACGCCTCGGACCAGGAGGT GCTGGACATGCACGGTTTCCTGCGCCAGGCGCTCTGCCGCCTGCGCCAGGAGGAGCCCCAGAGC CTGCAAGCTGCCGTGCGCACCGATGGCTTCGACGAGTTCAAGGTGCGCCTGCAGGACCTCAGCTC TTGCATCACCCAGGGGAAAGCCATTGAGACCCAGAGCAGCAGTTCTGAagagatagtgcccagccctccctcgc caccccctctaccccgcatctacaagccttgctttgtctgtcaggacatgctagttattgctcagcggccgc
*cABL*	TAATACGACTCACTATAGGGGTGCGTGAGAGTGAGAGCAGTCCTGGCCAGAGGTCCATCTCGCT GAGATACGAAGGGAGGGTGTACCATTACAGGATCAACACTGCTTCTGATGGCAAGCTCTACGTC TCCTCCGAGAGCCGCTTCAACACCCTGGCCGAGTTGGTTCATCATCATTCAACGGTGGCCGACG GGCTCATCACCACGCTCCATTAATGCTAGTTATTGCTCAGCGGCCGC

translocation breakpoint is discriminated by lowercase and uppercase characters

For the sequences of fusion genes, upper and lower cases were used to distinguish the portion of each gene.

### Eprobe mediated RT-qPCR

**Fig 1:**
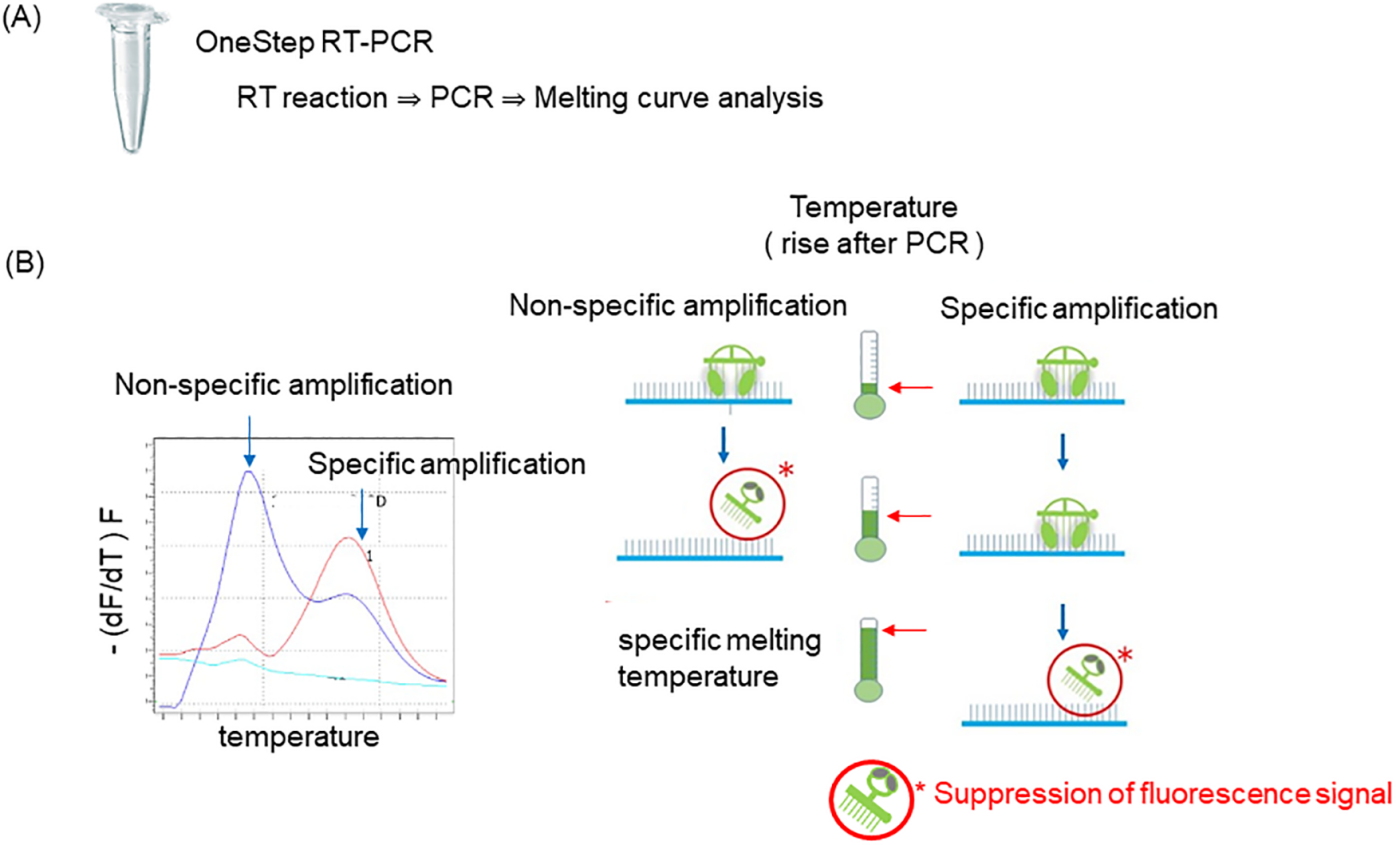
Schematic illustration of polymerase chain reaction (PCR) Eprobe melting curve analysis. (A) Eprobe mediated reverse transcription quantitative PCR (RT-qPCR). Fusion transcripts were amplified in a single-tube setting by using standard PCR primers with Eprobe. (B) Melting curve analysis. Eprobe binds to complementary DNA with higher affinity than normal oligonucleotides by exploiting cationic dye moieties, leading to a competitive effect in primer annealing and extension. Accordingly, melting curve analysis can be used to confirm that a single amplicon has been detected by the Eprobe.

Primers and Eprobes were designed for the following fusion transcripts using Edesign: major and minor *BCR-ABL1*, *PML-RARA* (bcr1, bcr2, and bcr3) and *RUNX1-RUNX1T1*.[17] *cABL* was used as a housekeeping gene to determine the amplifiability and quality of the cDNAs.[18] As the reference for the assay of major *BCR-ABL*, ARQ IS Calibration Panel^™^ was also employed (Lot# DL14-146). Eprobes were obtained from DNAFORM (Yokohama, Japan). The sequences for all Eprobes and PCR primers for the assays are described in Table 2. A schematic illustration of the single-tube Eprobe RT-qPCR assay is shown in Fig 1. In each reaction tube, 1 μg of total RNA was reverse-transcribed and subjected to PCR amplification in a 20-μL reaction volume containing 1× OneStep RT-PCR Buffer, 400 μM dNTPs, 0.1 μM forward primer, 0.5 μM reverse primer, 0.4 μM Eprobe, and 0.2 μL enzyme mix (including 20 units of reverse transcriptase [RT] and 0.5 units of DNA polymerase). RT-qPCR was performed in LightCycler 480 (Roche Diagnostics, Mannheim, Germany) using the following parameters: 45°C for 15 min (RT step), followed by 95°C for 10 min (for inactivation of RT and activation of Hot Start DNA polymerase), and 50 PCR cycles at 95°C for 10 s (denaturing), 55°C for 40 s (annealing), and 72°C for 40 s (extension). The fluorescence signals were obtained with a SYBR Green I filter set (excitation at the wave length of 483 nm; and emission, 533 nm). Negative controls that do not contain RNA templates were utilized in each run to exclude contaminations. Standard curves for RT-qPCRs were generated by analyzing the crossing point (Cp) values. Calculation of the Cp value from each amplification curve and extraction of the standard curve were performed using ‘Abs Quant/Fit Point’ analysis of LightCycler 480 software release 1.5.1.62 SP3, version 1.5.1.62.

**Table 2:** PCR primer and Eprobe sequences.

Major *BCR-ABL1*	
forward primer	5'-TGACCAACTCGTGTGTGAAACTC-3'
reverse primer	5'-CACTCAGACCCTGAGGCTCAA-3'
Eprobe	5'-CC**T**TCAGCGGCC-3'
minor *BCR-ABL1*	
forward primer (old)	5'-CTGGCCCAACGATGGCGA-3'
reverse primer (old)	5'-CAACGAGCGGCTTCACTCA-3'
forward primer (new, F06)	5'-CGGGCAGATCTGGCCCAA-3'
reverse primer (new, F06)	5'-TAGACTGAAACTCGGAGTCCCA-3'
probe	5'-CC**T**TCAGCGGCC-3'
*RUNX1-RUNX1T1*	
forward primer	5'-GTCGAAGTGGAAGAGGGAAA-3'
reverse primer	5'-CACAGGTGAGTCTGGCATTG-3'
probe	5'-GAAATCG**T**ACTGAGAAGCA-3'
*PML-RARA*	
forward primer (bcr1)	5'-TCTTCCTGCCCAACAGCAA-3'
forward primer (bcr2)	5'-CCCAGGGACCCTATTG-3'
forward primer (bcr3)	5'-CTTGCATCACCCAGGGGAAA-3'
reverse primer	5'-AGGGAGGGCTGGGCACTATC-3'
probe	5'-CCA**T**TGAGACCCAGAGC-3'
*cABL*	
forward primer	5'-CGAAGGGAGGGTGTACCATTA-3'
reverse primer	5'-CAACTCGGCCAGGGTGTT-3'
probe	5'-GCTCTACGTC**T**CCTC-3'

### Melting curve analyses associated with Eprobe qRT-PCR

Melting curve experiments with real-time fluorescent monitoring were subsequently performed after Eprobe qRT-PCRs to exclude non-specific reactions (false-positives) as described elsewhere.[13, 19] The amplified products were treated with the PCR protocols: heating at 95°C for 15 s, cooling to 40°C, holding at 40°C for 1 min, and then slowly heating again to 95°C at a ramp rate of 0.11°C/s. To obtain the melting curve signals, continuous fluorescence acquisition (483/533 nm, five times per °C) was carried out for the temperature elevating period from 40 to 95°C.

### TaqMan RT-qPCR

The assays developed in our laboratory were previously validated in the clinical testing. The fusion transcript-specific primers based on the sequences of the oligonucleotides were developed according to the published methods with minor modifications.[15] *cABL* was used as a housekeeping gene to confirm the amplifiability and quality of the cDNA.[15] Sequences for all TaqMan PCR primers and probes are described in Table 3. Samples from our archive of clinical specimens were chosen based on positive RT-PCR results for the tested fusion genes, sufficient RNA to perform the Eprobe RT-qPCR assay, and the ability to perform additional assays as warranted for the clinical follow-up of each individual patient.

**Table 3:** PCR primer and oligonucleotide probe sequences of standard diagnostic TaqMan PCR.

Major *BCR-ABL1*	
forward primer	5'-TGACCAACTCGTGTGTGAAACTC-3'
reverse primer	5'-CACTCAGACCCTGAGGCTCAA-3'
probe	FAM-CCCTTCAGCGGCCAGTAGCATCTGA-TAM
minor *BCR-ABL1*	
forward primer	5'-CTGGCCCAACGATGGCGA-3'
reverse primer	5'-CAACGAGCGGCTTCACTCA-3'
probe	FAM-CCCTTCAGCGGCCAGTAGCATCTGA-TAM
*RUNX1-RUNX1T1*	
forward primer	5'-GTCGAAGTGGAAGAGGGAA-3'
reverse primer	5'-CAATGCCAGACTCACCTGTG-3'
probe	FAM-AACCCACCGCAAGTCGCCAC-TAM
*PML-RARA*	
forward primer (F1)	5'-TCTTCCTGCCCAACAGCAA-3'
forward primer (F2)	5'-CCCAGGGACCCTATTG-3'
forward primer (F3)	5'-CATGCACGGTTTCCTG-3'
reverse primer	5'-AGGGAGGGCTGGGCACTATC-3'
probe	FAM-CCATTGAGACCCAGAGCAGCAGCTCTGA-TAM
*cABL*	
forward primer	5'- CGAAGGGAGGGTGTACCATTA -3'
reverse primer	5'- CAACTCGGCCAGGGTGTT -3'
probe	FAM-CTTCTGATGGCAAGCTCTACGTCTCCTCC-TAM

### Statistical analysis

Statistical analyses were performed using Microsoft Excel 2016 (Microsoft Corp., Tokyo, Japan) software. To investigate correlation between major and minor *BCR/ABL1* expression level for individual patient sample, we performed linear regression by least squares method. Significance of obtained correlation coefficient (*r*) was evaluated by t-test with *n*-2 degree of freedom. Values of *p*< 0.05 were considered statistically significant.

## Results

### Evaluation of performance of the Eprobe RT-qPCR assay using synthetic RNA

Performance of the Eprobe mediated RT-qPCR assay of major and minor *BCR-ABL1*, *RUNX1-RUNX1T1*, and *PML-RARA* (bcr1, bcr2, and bcr3) were evaluated using synthesized RNA. First, the quantification capacity of the Eprobe RT-qPCR assay was evaluated based on linearity[20] by comparison with TaqMan RT-qPCR. Eprobe RT-qPCR demonstrated the performance equivalent to TaqMan RT-qPCR in all tested assays (S1 and S2 Figs).

To assess the lower limit of detection (LOD) and the limit of quantification (LOQ) of each fusion transcript by Eprobe RT-qPCR, serially diluted samples of the synthetic RNAs were tested 10 times at the concentrations of 5.2, 10.3, 20.8, and 31.3 copies/reaction, and three times at the concentrations of 62.5, 12.5 × 10^2^, 2.5 × 10^4^, 5.0 × 10^5^, and 1.0 × 10^7^ copies/reaction along with the negative samples. The LOD could be lowered to 10.4 copies/reaction in major *BCR-ABL1*, *RUNX1-RUNX1T1* and *PML-RARA* (bcr2), to 20.8 copies in *PML-RARA* (bcr1 and bcr3), and to 31.3 copies in minor *BCR-ABL1* (Table 4). The LOQ, defined as the detectable lowest copy number whose coefficient of variation was smaller than 10%, could be lowered to 20.8 copies in major *BCR-ABL1*, to 31.3 copies in *RUNX1-RUNX1T1* and *PML-RARA* (bcr2), and to 62.5 copies in minor *BCR-ABL1* and *PML-RARA* (bcr1 and bcr3) (Table 5).

**Table 4:** The lower limit of detection (LOD) by Eprobe RT-qPCR.

	Target value (copies / reaction)								
	5.2	10.4	20.8	31.3	62.5	12.5 x10^2^	2.5x10^4^	5.0x10^5^	1.0x10^7^
Major *BCR-ABL1*	8/10*	**10/10**	10/10	10/10	3/3	3/3	3/3	3/3	3/3
minor *BCR-ABL1*	6/10	7/10	8/10	**10/10**	3/3	3/3	3/3	3/3	3/3
*RUNX1-RUNX1T1*	9/10	**10/10**	10/10	10/10	3/3	3/3	3/3	3/3	3/3
*PML-RARA* (bcr1)	6/10	8/10	**10/10**	10/10	3/3	3/3	3/3	3/3	3/3
*PML-RARA* (bcr2)	8/10	**10/10**	10/10	10/10	3/3	3/3	3/3	3/3	3/3
*PML-RARA* (bcr3)	8/10	7/10	**10/10**	10/10	3/3	3/3	3/3	3/3	3/3

* number of detected / tested sample

underlined; LOD

**Table 5:** The limit of quantification (LOQ) by Eprobe RT-qPCR; CV%.

	Target value (copies / reaction)								
	5.2	10.4	20.8	31.3	62.5	12.5 x10^2^	2.5x10^4^	5.0x10^5^	1.0x10^7^
Major *BCR-ABL1*	30.96*	34.77	**6.94**	8.78	2.39	0.28	0.47	0.77	1.00
minor *BCR-ABL1*	36.53	37.64	13.21	15.61	**8.40**	0.47	0.93	0.77	2.52
*RUNX1-RUNX1T1*	25.30	9.83	12.96	**6.37**	5.88	2.44	3.04	6.57	1.58
*PML-RARA* (bcr1)	15.80	22.28	14.54	17.53	**5.00**	3.65	5.62	2.03	2.50
*PML-RARA* (bcr2)	73.00	23.37	11.38	**8.20**	5.57	1.66	6.20	3.79	0.74
*PML-RARA* (bcr3)	28.91	12.34	19.20	14.61	**3.74**	0.48	1.71	0.68	0.67

* CV % (percent of coefficient of variation)

underlined; CV % of LOQ target value

### Performance of Eprobe mediated RT-qPCR in primary samples

Eprobe RT-qPCR detected the same fusion transcripts as our existing laboratory-developed TaqMan RT-qPCR in all positive cases.[15] Complete concordance was also achieved in the negative cases. The results were consistent with cytogenetics (Table 6). There were concerns about the sensitivity of transcript levels; all fusion genes tested by the Eprobe and TaqMan RT-qPCR techniques were detected at similar amplification levels except *PML-RARA* bcr3. The Eprobe RT-qPCR assay with the bcr3-specific forward primer set exhibited higher amplification than TaqMan RT-qPCR that shared the bcr1 primer set for bcr3 detection (Tables 2 and 3, S1 Table). The comparison of Taq-Man and Eprobe RT-qPCR in the measurement of major *BCR-ABL* was also performed using ARQ IS Calibration Panel (Armored RNA Quant IS Calibrator Panel (ARQ IS), Asuragen, Inc., Austin, TX, USA). The results confirmed the high sensitivity of Eprobe assays (S2 Table).

**Table 6:** Summary of comparison of Taq-Man and Eprobe RT-qPCR of primary samples.

In-house Taq-Man PCR test result	Sample number	Eprobe RT-qPCR		Cytogenetics concordance
		Positive	Negative	
Major *BCR-ABL1*	19			
positive	10	10/10	0/10	6/6 (4; N.A.)
negative	9	0/9	9/9	
minor *BCR-ABL1*	14			
positive	4	4/4	0/4	2/2 (2; N.A.)
negative	10	0/10	10/10	
*RUNX1-RUNX1T1*	21			
positive	12	12/12	0/12	8/8 (4; N.A.)
negative	9	0/9	9/9	
*PML-RARA*	13			
positive (bcr1)	3	3/3	0/3	3/3
positive (bcr3)	2	2/2	0/2	0/2
negative	8	0/8	8/8	

N.A., not applicable.

### Detecting low-level minor *BCR-ABL1* in major and minor *BCR-ABL1* double-positive CML cases

**Fig 2:**
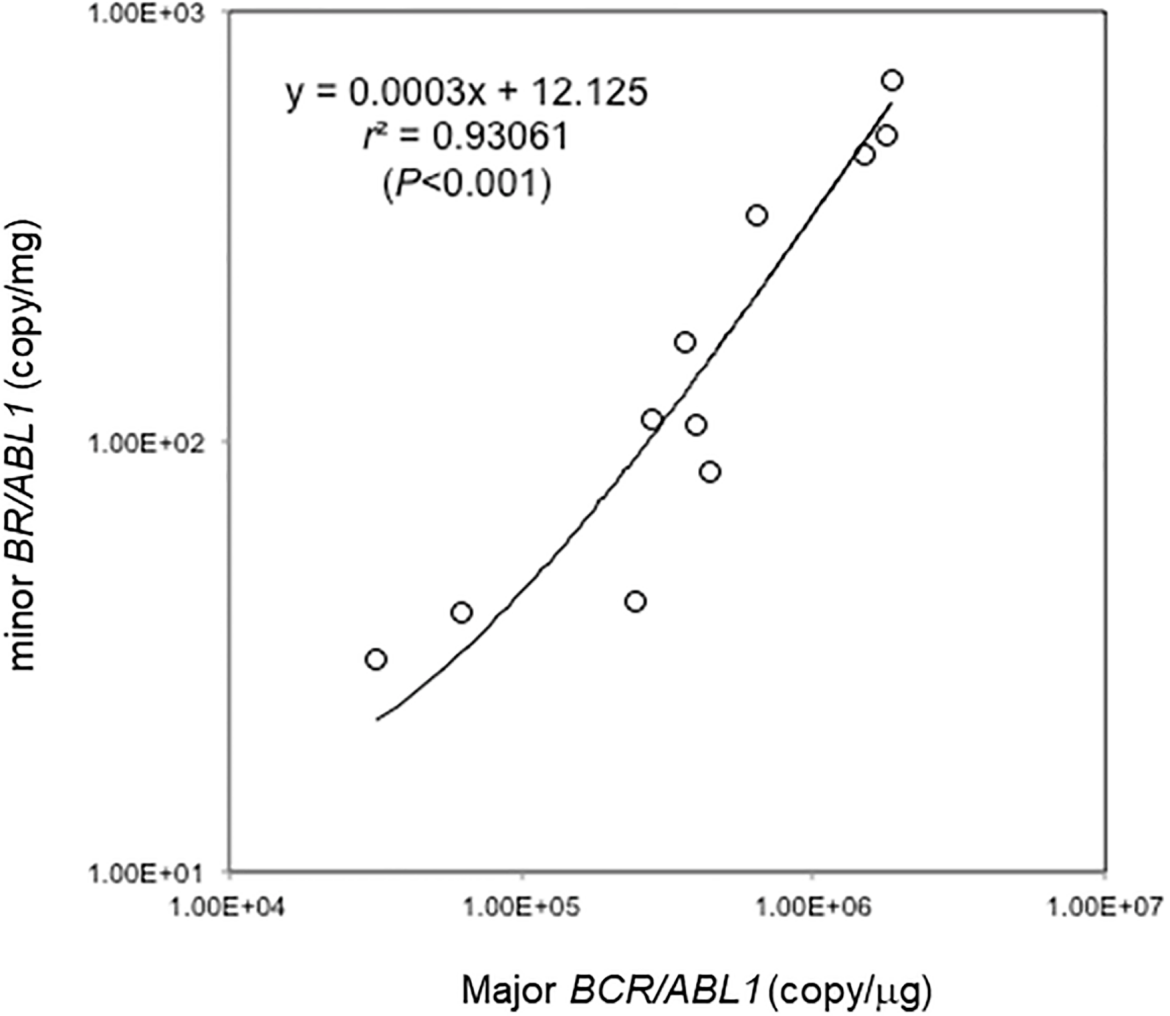
Correlation of major and minor *BCR-ABL1* mRNA expression levels in double-positive chronic myeloid leukemia (CML) cases. Major and minor *BCR-ABL1* mRNA expression levels were compared in the minor *BCR-ABL1* co-expressed specimens obtained from major *BCR-ABL1*-positive CML cases (11/19, 58%) by TaqMan RT-qPCR.

**Fig 3:**
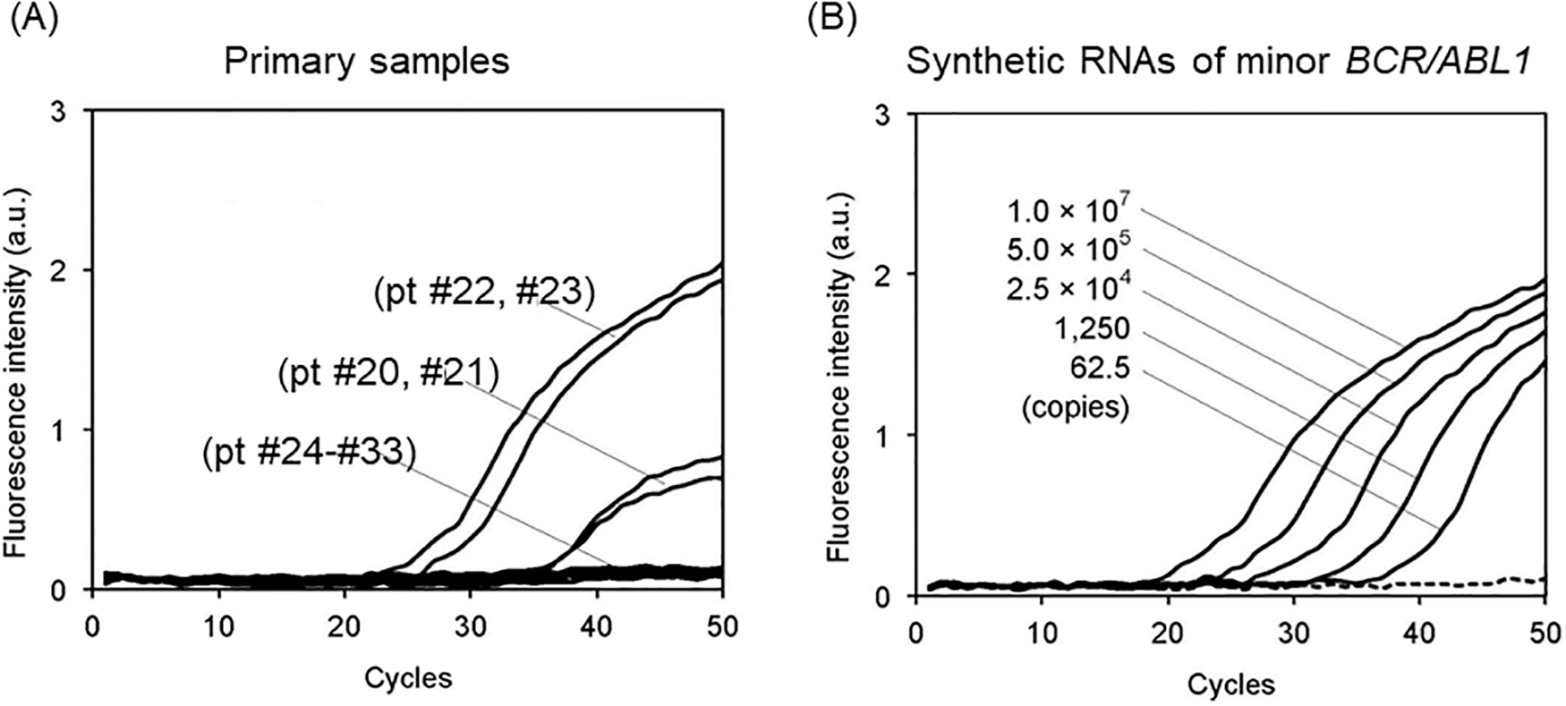
Abnormal amplification curves in primary samples with low transcript levels of minor *BCR-ABL1*. (A) The abnormally low degree of PCR amplification in primary samples with low-level minor *BCR-ABL1* transcripts (pt #20 and #21, S1 Table) detected by Eprobe mediated RT-qPCR (performed with the old primer sets indicated in Table 2). (B) Normal amplification curve lines in synthetic minor *BCR-ABL1* standard RNA detected by Eprobe mediated RT-qPCR with the old primer set (Table 2).

Low level of minor *BCR-ABL1* transcripts can be detected in some CML patients in whom major *BCR-ABL1* transcripts are dominantly expressed.[21] Consistent with the previous report,[21] concomitant presence of the minor *BCR-ABL1* was detected in 11 of 19 samples (58%) obtained from the CML cases in which major *BCR-ABL1* were detected. In these cases, the copy numbers of the major and minor *BCR/ABL1* were somehow positively correlated despite the transcript levels of minor *BCR/ABL1* were extremely low (Fig 2). S2 Table indicates the copy numbers for each sample. As shown in Fig 3A, an abnormally low sloped amplification curve line was observed during the detection of low-level minor *BCR-ABL1* transcripts by Eprobe RT-qPCR (performed with the old primer set indicated in Table 2). Similar results were obtained in the same samples using TaqMan RT-qPCR (data not shown). On the contrary, when the synthetic minor *BCR-ABL1* standards were measured, the fluorescence intensity was proportional to the amount of RNAs even at very low levels (Fig 3B).

**Fig 4:**
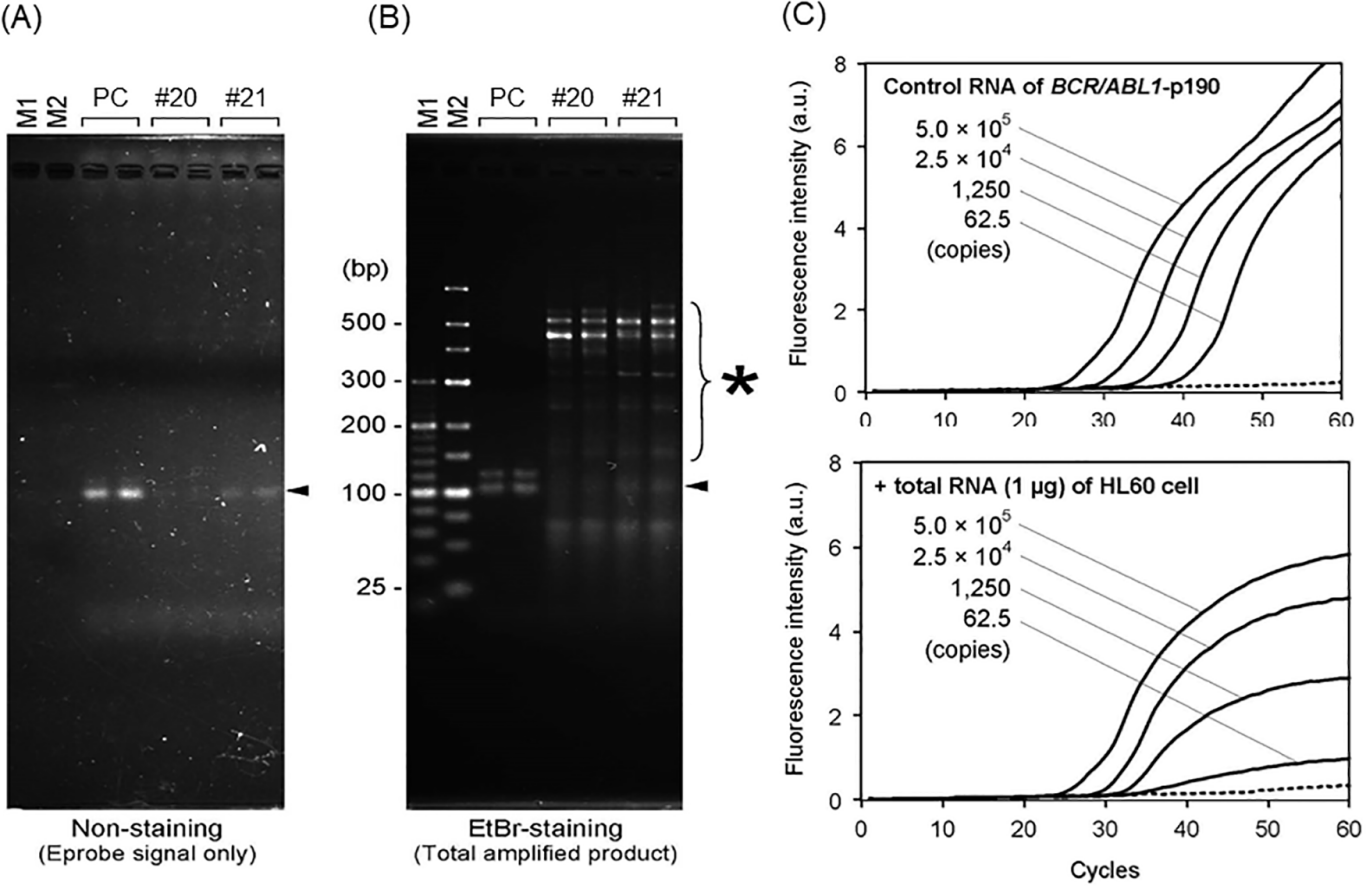
Non-specific amplification of co-existing RNA interferes the detection of minimal expression of minor *BCR-ABL1*. (A) Eprobe fluorescent signals of the Eprobe RT-qPCR products detected by agarose gel (1.5%) electrophoresis. PC depicts positive control that contains synthetic minor *BCR-ABL1* transcripts (1.0 × 10^7^ copies/reaction); #20 and #21, primary samples with low expression of minor *BCR-ABL1* transcripts, respectively. (B) Same series of samples as shown in panel (A) stained with ethidium bromide. (C) Amplification curve lines of serially diluted synthetic minor *BCR-ABL1*-spiked-in samples. *Copy numbers of spiked-in synthetic minor *BCR-ABL1*/reaction in total RNA of HL60 cells (1μg) has been shown.

To investigate this discrepancy, we performed electrophoresis experiments. The Eprobe RT-qPCR products of low-level minor *BCR-ABL1*-positive samples (pt #20 and #21 shown in Fig 3) were analyzed using agarose gel electrophoresis. Without staining, these samples showed a single band of Eprobe fluorescence at the expected molecule size indicating that the segment containing the minor *BCR-ABL1* transcripts were bound to the Eprobe (indicated by the black arrow in Fig 4A). However, when the gel was stained with ethidium bromide, several non-specific amplicons were observed (marked with * in Fig 4B) indicating that the non-specific PCR reactions might cause a limitation to detect extremely low copy numbers of minor *BCR-ABL1* transcripts with Eprobes in actual patient samples. These findings in patient samples were confirmed by Eprobe RT-qPCRs using various amounts of synthetic minor *BCR-ABL1* transcripts in the absence or presence of total RNAs extracted from HL60 acute myeloid leukemia cell lines. The HL60 cell lines do not express minor *BCR-ABL1*, which mimics the conditions of RT-qPCRs using the patient samples. Fig 4C upper panel shows representative amplification curves using sequentially diluted minor *BCR-ABL1* transcripts. Above the copy number of 62.5, the estimated Ct values were proportional to the actual copy numbers. On the contrary, in the presence of total RNAs of HL60 cells, the amplification curves became unproportional to the copy numbers (Fig 4C lower panel).

**Fig 5:**
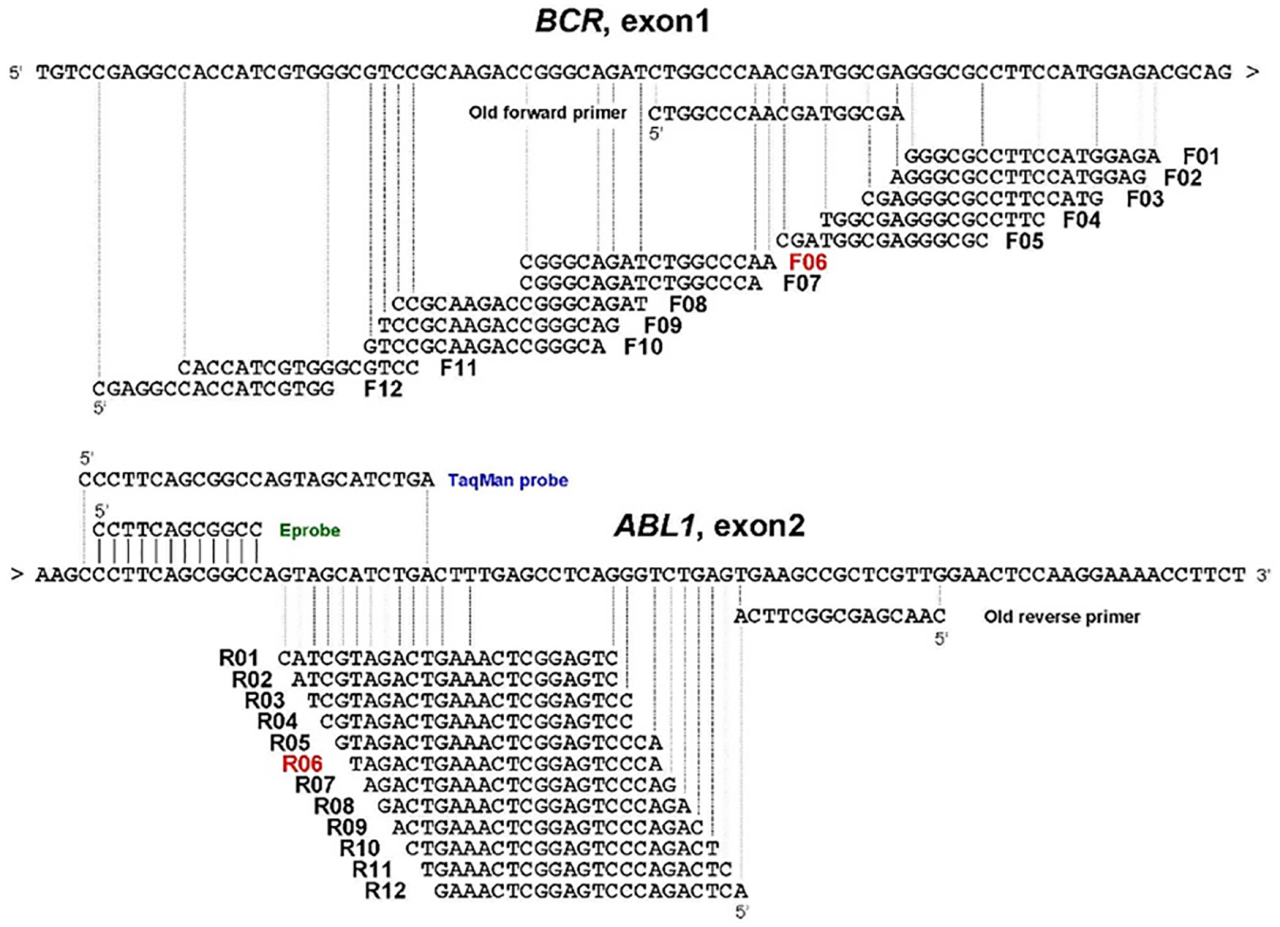
New primer candidates of minor *BCR-ABL1* for Eprobe mediated RT-qPCR.

**Fig 6:**
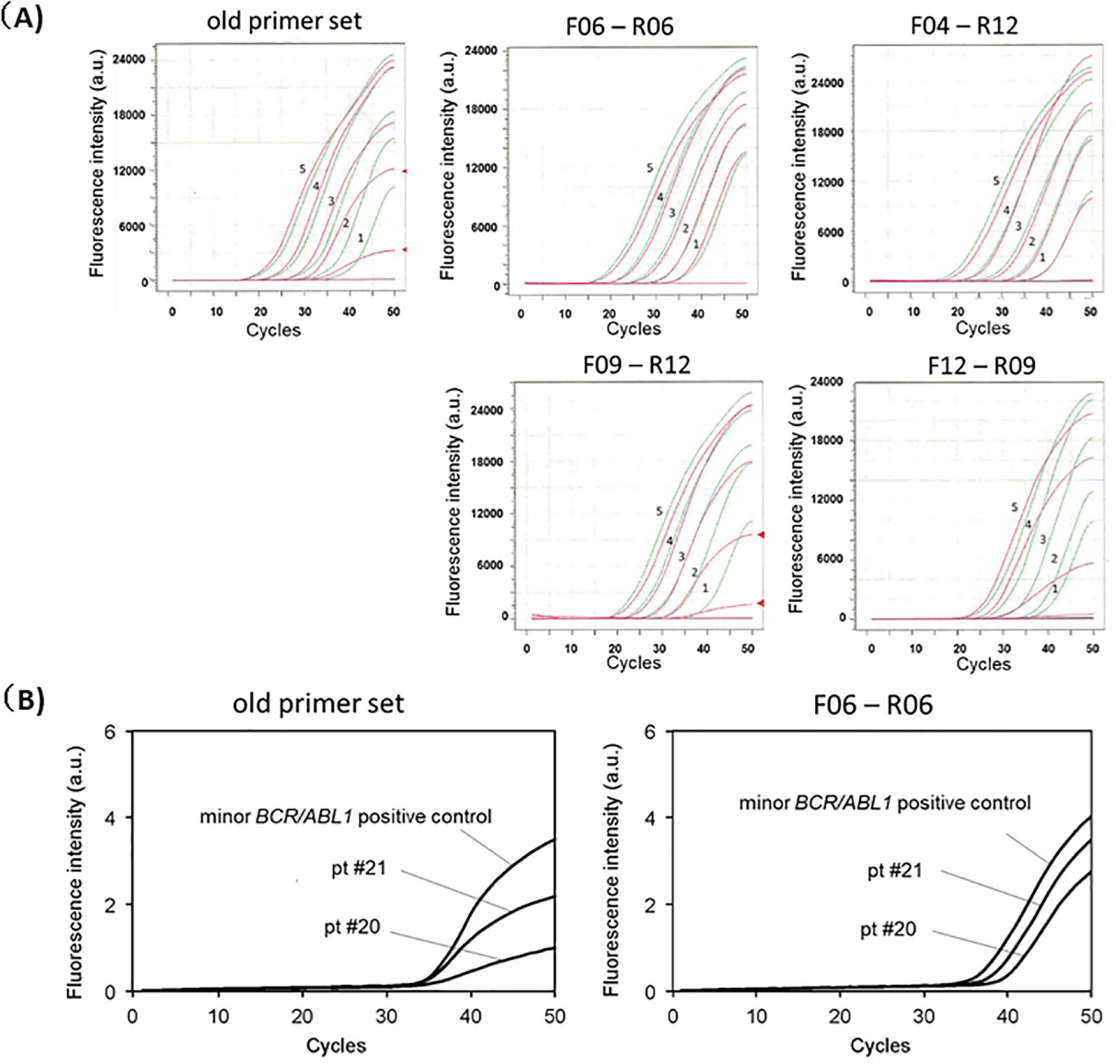
Amplification curves of the Eprobe mediated RT-qPCR with various combinations of the primer sets using synthetic RNAs and patient samples. (A) Eprobe RT-qPCR amplification curve lines of minor *BCR-ABL1* using various sets of primers. Samples of known concentration were used: (1) synthetic minor *BCR-ABL1* RNA (63 copies/reaction)-spiked-in HL60 total RNA; (2) 1,250 copies/reaction spiked-in; (3) 2.5×10^4^ copies/reaction spiked-in; (4) 5.0×10^5^ copies/reaction spiked-in; (5)1.0×10^7^ copies/reaction spiked-in. Red lines depict synthetic minor *BCR-ABL1* RNA with HL60 total RNA; green lines, synthetic minor *BCR-ABL1* RNA without HL60 total RNA. (B) Ampification curves in major and minor *BCR-ABL1* dual-positive CML primary samples.

One of the possible explanations for these observations is that non-specific PCR reaction associated with unknown genes might have taken place when the living cells were used. In other words, the PCR primer sets, we thought were specific, were not true, resulting in unexpected consuming of the PCR primers by unknown genes. If this is the case, we need to use different primer sets that are truly specific to detect the minor *BCR-ABL1* transcripts. In order to do so, we redesigned a series of primers (11 forward primers and 12 reverse primers) for the Eprobe RT-qPCR assay (Fig 5). These sets of primers were evaluated using the synthetic minor *BCR-ABL1* RNA-spiked-in HL60 total RNAs. Fig 6A shows the amplification curves using various combinations of the primers. The R6 forward and reverse primers showed the most prominent specific amplification curve lines. This primer sets, but not the old primers, successfully detected low-level minor *BCR-ABL1* transcripts in the samples obtained from patient #20 and #21 (Fig 6B).

### Melting curve analysis by Eprobe

In contrast to the TaqMan probe, the Eprobe is not digested during PCR so that it keeps generating adequate fluorescence signals that can be used for post-PCR melting curve analyses. Eprobes bind to the template DNA during annealing step and the fluorescent signals are detected, then they dissociate from the template again in a temperature-dependent manner. Melting curve analysis by Eprobe successfully detected specific reactions in all the tested primary samples. It enabled us to exclude off-target amplification (S3 Fig).

## Discussion

In the CAP/ASH guideline for initial diagnosis of acute leukemias, prompt and accurate identification of leukemia-related fusion transcripts are strongly recommended since these abnormal transcripts can be targeted by specific pharmacotherapies.[7] In the present study, we successfully developed a new assay platform utilizing Eprobes, which enables us to perform standardized protocols quickly, accurately, and cost-effectively.

Fluorescence probe technologies such as Eprobes and TaqMan probes exert high specificity for target PCR amplicon detections by suppressing false-positive signals generated from non-specific products. Currently, however, TaqMan probe assays are performed based upon home-brewed methods at each institution, which may cause inconsistency in the treatments. In addition, it is a time-consuming and expensive procedure. The Eprobe RT-qPCR assay system has overcome these disadvantages of TaqMan RT-qPCR assays. To validate the Eprobe technology, we compared various parameters between these two assays. For example, low-level transcripts of minor *BCR-ABL1* are often detected in major *BCR-ABL1*-positive CML cases.[21] We observed a decay in the amplification curve line caused by lower copy numbers of target RNA in the clinical samples, while the decay did not occur in case of generating the standard curve using with artificial RNA including target RNA only. The decay was caused by the non-specificity of the PCR primers, which was proved by the spike-in experiment of target RNA into target negative total RNA as alternatives for target RNA positive sample. Because the primers contributed to amplification of non-specific products, especially in case of low-expression level of target RNA, the amplification ratio of the target products decreased and the plateau level of the fluorescence signal decreased. In these cases, altered amplification curve lines caused by low expression levels of target products led to generating inaccurate Ct values.

The Eprobe RT-qPCR assay was designed to be applicable to a single test and to multiple screenings. The main advantage of this Eprobe RT-qPCR assay lies in its simplicity; allows us to reduce non-specific reactions by combining amplification and melting curve analysis within a single-tube reaction, thus avoiding gel electrophoresis and sequencing, and contributes to a rapid and an accurate diagnosis. The implementation of synthetic positive control RNAs within the Eprobe RT–qPCR assay provides routine performance control of the tests and guarantees high-quality standards.

The initial workup and evaluation of acute leukemias have become increasingly complex over the past decades since so many new genetic abnormalities have been discovered, which might affect the prognosis and might be critical to determine the therapeutic strategies.[7, 22, 23] In the future, newer diagnostic technologies such as genome-wide next-generation sequencing and high-density microarrays have become extremely valuable for global gene expression profiling of patients. However, the CAP/ASH guidelines do not recommend the use of these global gene expression analyses as standard methods because there are still many genetic abnormalities whose pathogenic roles are unknown.[7] For the current clinical practice, the analysis of small subsets of targets is required in hematopathology laboratories, and the Eprobe RT-qPCR assay system presented herein is not intended to cover all possible transcript variants, instead being limited to the most frequent and prognostically relevant fusion genes.[8]

It is important to note the limitations of this study. First, this is a single-center study and included a relatively small number of samples. Second, only limited types of leukemias were covered. Currently, we plan to expand our study to incorporate more data from multiple centers for future studies. An “Eprobe leukemia panel kit” based on this Eprobe mediated RT-qPCR assay, equipped with synthetic quantitative standard RNAs with strict quality control and additional targets including *CBFb-MYH11*, *ETV6-AML1*, *MLL-AF4*, and *MLL-AF9* rearrangements is currently under development.

In conclusion, the Eprobe mediated assay system demonstrated an ability for accurate and rapid RT–qPCR detection of fusion transcripts in certain types of leukemias, thus serving as a useful molecular diagnostic method. Widespread implementation of this method at hospital clinical laboratories may contribute to high-quality test results and efficient quality control.

## Supporting information

S1 Table Comparison of Taq-Man and Eprobe RT-qPCR for fusion transcript detection of primary samples. N.D., not detected; N.A., not applicable; *detected PML-RARA (bcr1); **detected PML-RARA (bcr3).
(DOCX)

S2 Table Comparison of Taq-Man and Eprobe RT-qPCR for ARQ IS Calibrator Panel TM e14a2. % BCR-ABL/cABL*: calculated the mean of % *BCR-ABL/cABL* N.D.; not detected. Cp; crossing point.
(DOCX)

S3 Table Raw data of Fig 2. (DOCX)

S1 Fig Performance of the Eprobe RT-qPCR using synthetic *BCR-ABL1* fusion transcripts. Eprobe mediated RT-qPCR experiments were performed with different concentrations of standard RNA. Amplification curve lines and PCR efficiency plots (threshold cycle [Ct] values plotted against the logarithm of standard RNA concentrations) are shown. RNA concentrations used for the Eprobe-based reaction were 62.5, 12.5 × 10^2^, 2.5 × 10^4^, 5.0 × 10^5^, and 1.0 × 10^7^ copies/reaction.
(JPG)

S2 Fig Performance of the TaqMan RT-qPCR using synthetic *BCR-ABL1* fusion transcripts. TaqMan RT-qPCR experiments were performed by the exact same methods described in S1 Fig. RNA concentrations used for the TaqMan probe-based reaction were 10, 10^2^, 10^3^, 10^4^, 10^5^, 10^6^, 10^7^, and 10^8^ copies/reaction.
(JPG)

S3 Fig Melting curve analysis of amplification products. Results of melting curve analyses are shown as the first derivative curve by plotting fluorescence (*F*) variation (-[*dF/dT*]*F*) against temperature (*T*), which was derived from Eprobe mediated RT-qPCR products of the indicated leukemia-related fusion transcripts. Red arrow indicates the melting peak of each respective product. The theoretical Tm value of each targeted Eprobe is shown as follows: major and minor *BCR-ABL1*, 65.0°C; *RUNX1-RUNX1T1*, 62.5°C; *PML-RARA* (bcr1, bcr2, or bcr3), 64.7°C. Each theoretical Tm value was estimated using the ECHO/DNA Thermodynamics method^19^.
(JPG)
