# Supplementary material for: Eprobe mediated RT-qPCR for the detection of leukemia-associated fusion genes
Source: PLoS One. 2018 Oct 3;13(10):e0202429. doi: 10.1371/journal.pone.0202429 (PMC6169845; doi:10.1371/journal.pone.0202429)
Supplement: S1 Table — N.D., not detected; N.A., not applicable; *detected PML-RARA (bcr1); **detected PML-RARA (bcr3). (DOCX) [file pone.0202429.s001.docx]

**S1 Table. Comparison of Taq-Man and Eprobe RT-qPCR for fusion transcript detection of primary samples**

| Major *BCR-ABL1* |  |  |  |  |  |  |
| --- | --- | --- | --- | --- | --- | --- |
| patient # | *BCR-ABL1* p210 (copy / ugRNA) | | *cABL (copy / ugRNA)* | | *BCR-ABL1 p210 / cABL* ratio | |
|  | Eprobe | Taq-Man | Eprobe | Taq-Man | Eprobe | Taq-Man |
|  |  |  |  |  |  |  |
| 1 | 2.24E+03 | 1.20E+03 | 9.37E+05 | 7.85E+06 | 2 .39E-01 | 1.53E-02 |
| 2 | 1.50E+04 | 3.92E+03 | 6.42E+05 | 5.11E+06 | 2.33E+00 | 7.67E-02 |
| 3 | 3.33E+01 | 1.71E+01 | 9.89E+05 | 5.63E+06 | 3.37E-03 | 3.04E-04 |
| 4 | 9.85E+01 | 5.09E+01 | 1.27E+06 | 4.48E+06 | 7.73E-03 | 1.14E-03 |
| 5 | 4.12E+01 | 7.68E+00 | 8.46E+05 | 3.02E+06 | 4.87E-03 | 2.54E-04 |
| 6 | 3.02E+02 | 7.51E+01 | 1.07E+06 | 6.08E+06 | 2.83E-02 | 1.24E-03 |
| 7 | 3.03E+01 | 2.33E+01 | 1.08E+06 | 6.35E+06 | 2.80E-03 | 3.67E-04 |
| 8 | 2.98E+04 | 1.33E+04 | 8.04E+05 | 2.47E+06 | 3.71E+00 | 5.38E-01 |
| 9 | 3.72E+02 | 3.34E+02 | 4.78E+05 | 1.29E+06 | 7.78E-02 | 2.59E-02 |
| 10 | 7.06E+05 | 6.83E+05 | 1:94E+06 | 1.34E +07 | 3.64E+01 | 5.10E+00 |
| 11 | N.D. | N.D. | 7.08E+05 | 5.54E+06 | N.A. | N.A. |
| 12 | N.D. | N.D. | 5.05E+05 | 2.53E+06 | N.A. | N.A. |
| 13 | N.D. | N.D. | 4.35E+05 | 2.31E+06 | N.A. | N.A. |
| 14 | N.D. | N.D. | 7.36E+05 | 3.14E+06 | N.A. | N.A. |
| 15 | N.D. | N.D. | 4.83E+05 | 1.71E+06 | N.A. | N.A. |
| 17 | N.D. | N.D. | 7.06E+05 | 6.70E+06 | N.A. | N.A. |
| 18 | N.D. | N.D. | 1.23E+06 | 8.28E +06 | N.A. | N.A. |
| 19 | N.D. | N.D. | 8.16E+05 | 6.95E+06 | N.A. | N.A. |
|  |  |  |  |  |  |  |
| minor *BCR-ABL1* |  |  |  |  |  |  |
| patient # | *BCR-ABL1* p190 (copy / ugRNA) | | *cABL (copy / ugRNA)* | | *BCR-ABL1* p190 */ cABL* ratio | |
|  | Eprobe | Taq-Man | Eprobe | Taq-Man | Eprobe | Taq-Man |
|  |  |  |  |  |  |  |
| 20 | 1.85E+02 | 1.71E+02 | 1.48E+06 | 7.08E+06 | 2.42E-03 | 1.25E-02 |
| 21 | 3.39E+02 | 6.90E+02 | 5.21E+06 | 2.41E+07 | 2.86E-03 | 6.50E-03 |
| 22 | 2.66E+05 | 1.94E+06 | 1.88E+06 | 1.46E+07 | 1.33E+01 | 1.41E+01 |
| 23 | 5.99E+05 | 1.03E+06 | 4.78E+ 06 | 1.10E+07 | 9.36E+00 | 1.25 E+01 |
| 24 | N.D. | N.D. | 5.22E+05 | 3.89E+06 | N.A. | N.A. |
| 25 | N.D. | N.D. | 1.19E+06 | 6.85E+06 | N.A. | N.A. |
| 26 | N.D. | N.D. | 1.17E+06 | 3 .96E+06 | N.A. | N.A. |
| 27 | N.D. | N.D. | 1.26E+06 | 1.16E+07 | N.A. | N.A. |
| 28 | N.D. | N.D. | 2.09E+06 | 3.59E+06 | N.A. | N.A. |
| 29 | N.D. | N.D. | 2.85E+06 | 6.70E+06 | N.A. | N.A. |
| 30 | N.D. | N.D. | 1.08E+06 | 5 .73E+06 | N.A. | N.A. |
| 31 | N.D. | N.D. | 1.08E+06 | 6.70E+06 | N.A. | N.A. |
| 32 | N.D. | N.D. | 1.23E+06 | 8.28E +06 | N.A. | N.A. |
| 33 | N.D. | N.D. | 8.16E+05 | 6.95E+06 | N.A. | N.A. |
|  |  |  |  |  |  |  |
| *RUNX1-RUNX1T1* | |  |  |  |  |  |
| patient # | *RUNX1-RUNX1T1* (copy / ugRNA) | | *cABL (copy / ugRNA)* | | *RUNX1-RUNX1T1 / cABL* ratio | |
|  | Eprobe | Taq-Man | Eprobe | Taq-Man | Eprobe | Taq-Man |
|  |  |  |  |  |  |  |
| 34 | 1.73E+06 | 1.87E+06 | 7.13E+05 | 6.43E+06 | 2.43E+02 | 2.91E+01 |
| 35 | 5.18E+03 | 1.86E+03 | 8.87E+05 | 7.63E+06 | 5.84E-01 | 2.00E-02 |
| 36 | 1.65E+06 | 1.03E+06 | 1.52E+06 | 4.87E+06 | 1.08E+02 | 2.12E+01 |
| 37 | 2.01E+03 | 2.63E+03 | 1.41E+05 | 1.24E+06 | 1.43E+00 | 2.10E-01 |
| 38 | 1.50E+07 | 4.60E+06 | 3.40E+06 | 6.68E+06 | 4.40E+02 | 6.89E+01 |
| 39 | 1.39E+06 | 3.01E+06 | 7.52E+05 | 7.50E+06 | 1.85E+02 | 4.01E+01 |
| 40 | 6.39E+03 | 2.00E+03 | 5.93E+05 | 2.07E+06 | 1.08E+00 | 1.00E-01 |
| 41 | 9.85E+06 | 2.29E+06 | 2.70E+06 | 3.57E+06 | 3.65E+02 | 6.42E+01 |
| 42 | 1.86E+06 | 2.30E+06 | 6.45E+05 | 4.32E+06 | 2.89E+02 | 5.32E+01 |
| 43 | 4.08E+03 | 2.62E+03 | 5.56E+05 | 1.05E+07 | 7.35E-01 | 2.00E-02 |
| 44 | 3.68E+02 | 2.60E+02 | 8.48E+04 | 3.46E+06 | 4.35E-01 | 1.00E-02 |
| 45 | 1.62E+05 | 1.25E+05 | 9.71E+05 | 3.61E+06 | 1.67E+01 | 3.46E+00 |
| 46 | N.D | N.D. | 1.23E+06 | 1.13E+07 | N.A. | N.A. |
| 47 | N.D | N.D. | 3.77E+05 | 1.21E+07 | N.A. | N.A. |
| 48 | N.D | N.D. | 7.47E+04 | 7.65E+06 | N.A. | N.A. |
| 49 | N.D | N.D. | 5.87E+05 | 6.28E+06 | N.A. | N.A. |
| 50 | N.D | N.D. | 3.61E+05 | 4.12E+06 | N.A. | N.A. |
| 51 | N.D | N.D. | 2.41E+05 | 4.03E+06 | N.A. | N.A. |
| 52 | N.D | N.D. | 7.38E+04 | 5.21E+06 | N.A. | N.A. |
| 53 | N.D | N.D. | 1.39E+06 | 9.95E+06 | N.A. | N.A. |
| 54 | N.D | N.D. | 9.15E+05 | 1.22E+07 | N.A. | N.A. |
|  |  |  |  |  |  |  |
| *PML-RARA* | |  |  |  |  |  |
| patient # | *PML-RARA* (copy / ugRNA) | | *cABL (copy / ugRNA)* | | *PML-RARA / cABL* ratio | |
|  | Eprobe | Taq-Man | Eprobe | Taq-Man | Eprobe | Taq-Man |
|  |  |  |  |  |  |  |
| 55 | 6.05E+03 | 2.00E+03 | 1.45E+05 | N.A, | 4.18E+00 | N.A. |
| 56* | 2.53E+05 | 2.50E+04 | 4.90E+05 | 8.45E+06 | 5.17E+01 | 2.96E-01 |
| 57* | 2.63E+04 | 3.30E+03 | 1.75E+05 | 3.01E+06 | 1.50E+01 | 1.10E+00 |
| 58** | 4.10E+06 | 3.06E+03 | 1.70E+06 | 2.03E+06 | 2.41E+02 | 1.50E-01 |
| 59** | 2.50E+06 | 4.10E+03 | 9.90E+05 | 4.11E+06 | 2.53E+01 | 1.00E-01 |
| 60 | N.D | N.D. | 2.73E+06 | 1.95E+07 | N.A. | N.A. |
| 61 | N.D | N.D. | 2.05E+06 | 1.60E+07 | N.A. | N.A. |
| 62 | N.D | N.D. | 3.30E+05 | 5.75E+06 | N.A. | N.A. |
| 63 | N.D | N.D. | 5.79E+05 | 4.78E+06 | N.A. | N.A. |
| 64 | N.D | N.D. | 3.57E+05 | 4.57E+06 | N.A. | N.A. |
| 65 | N.D | N.D. | 3.58E+05 | 8.88E+06 | N.A. | N.A. |
| 66 | N.D | N.D. | 6.00E+03 | 2.70E+05 | N.A. | N.A. |
| 67 | N.D | N.D. | 4.90E+04 | 9.00E+04 | N.A. | N.A. |

N.D., not detected; N.A., not applicable; *detected PML-RARA (bcr1); **detected PML-RARA (bcr3)
