# Supplementary material for: Eprobe mediated RT-qPCR for the detection of leukemia-associated fusion genes
Source: PLoS One. 2018 Oct 3;13(10):e0202429. doi: 10.1371/journal.pone.0202429 (PMC6169845; doi:10.1371/journal.pone.0202429)
Supplement: S2 Table — % BCR-ABL/cABL*: calculated the mean of % BCR-ABL/cABL N.D.; not detected. Cp; crossing point. (DOCX) [file pone.0202429.s002.docx]

**S2_Table. Comparison of Taq-Man and Eprobe RT-qPCR for ARQ IS Calibrator Panel TM e14a2.**

| IS Panel (%) | Taq-Man | | | Eprobe | | |
| --- | --- | --- | --- | --- | --- | --- |
|  | *cABL* | *BCR-ABL1* | % *BCR-ABL/cABL** | *cABL* | *BCR-ABL1* | % *BCR-ABL/cABL** |
| Calibrator-1 (11.80) | 7.42.E+05 | 1.12.E+05 | 14.7 | 3.03.E+05 | 2.66.E+03 | 0.80 |
|  | 9.66.E+05 | 1.20.E+05 |  | 3.60.E+05 | 2.69.E+03 |  |
|  | 7.71.E+05 | 1.32.E+05 |  | 3.43.E+05 | 2.73.E+03 |  |
| Calibrator-2 (1.020) | 8.55.E+05 | 9.10.E+03 | 1.19 | 3.09.E+05 | 2.76.E+02 | 0.072 |
|  | 4.92.E+05 | 8.43.E+03 |  | 4.11.E+05 | 2.27.E+02 |  |
|  | 6.74.E+05 | 6.57.E+03 |  | 3.26.E+05 | 2.47.E+02 |  |
| Calibrator-3 (0.0740) | 6.74.E+05 | 8.33.E+02 | 0.108 | 3.20.E+05 | 1.97.E+01 | 0.0065 |
|  | 9.41.E+05 | 1.02.E+03 |  | 3.12.E+05 | 2.01.E+01 |  |
|  | 6.61.E+05 | 6.15.E+02 |  | 3.18.E+05 | 2.19.E+01 |  |
| Calibrator-4 (0.00430) | 9.54.E+05 | 2.45.E+02 | 0.022 | 4.21.E+05 | 1.29.E+00 | 0.00052 |
|  | 7.96.E+05 | 6.74.E+01 |  | 2.15.E+05 | 9.25.E-01 |  |
|  | 5.70.E+05 | 1.98.E+02 |  | 3.62.E+05 | 2.96.E+00 |  |
| Negative | 5.77.E+05 | N.D. | - | 3.35.E+05 | N.D. | - |
|  | 9.23.E+05 | N.D. |  | 2.74.E+05 | N.D. |  |
|  | 8.17.E+05 | N.D. |  | 2.91.E+05 | N.D. |  |
| Cp |  |  | 0.78 |  |  | 13.1 |

% *BCR-ABL/cABL**: calculated the mean of % *BCR-ABL/cABL*

N.D.; not detected.

Cp; crossing point
