# Supplementary material for: Eprobe mediated RT-qPCR for the detection of leukemia-associated fusion genes
Source: PLoS One. 2018 Oct 3;13(10):e0202429. doi: 10.1371/journal.pone.0202429 (PMC6169845; doi:10.1371/journal.pone.0202429)
Supplement: S3 Table — (DOCX) [file pone.0202429.s003.docx]

**S3 Table. Raw data of Fig 2.**

| Sample# | *cABL* | Major | minor |
| --- | --- | --- | --- |
| 30 | 8.65E+06 | 3.20E+04 | 3.12E+01 |
| 18 | 2.67E+06 | 6.26E+04 | 4.00E+01 |
| 14 | 6.30E+06 | 2.49E+05 | 4.23E+01 |
| 13 | 7.35E+06 | 4.47E+05 | 8.50E+01 |
| 38 | 1.41E+07 | 4.00E+05 | 1.10E+02 |
| 21 | 3.89E+06 | 2.82E+05 | 1.12E+02 |
| 23 | 7.08E+06 | 3.63E+05 | 1.71E+02 |
| 34 | 1.80E+07 | 6.36E+05 | 3.36E+02 |
| 51 | 4.75E+07 | 1.49E+06 | 4.61E+02 |
| 44 | 2.92E+07 | 1.77E+06 | 5.16E+02 |
| 32 | 2.41E+07 | 1.86E+06 | 6.90E+02 |
