# Supplementary figures and images for: Eprobe mediated RT-qPCR for the detection of leukemia-associated fusion genes
Source: PLoS One. 2018 Oct 3;13(10):e0202429. doi: 10.1371/journal.pone.0202429 (PMC6169845; doi:10.1371/journal.pone.0202429)

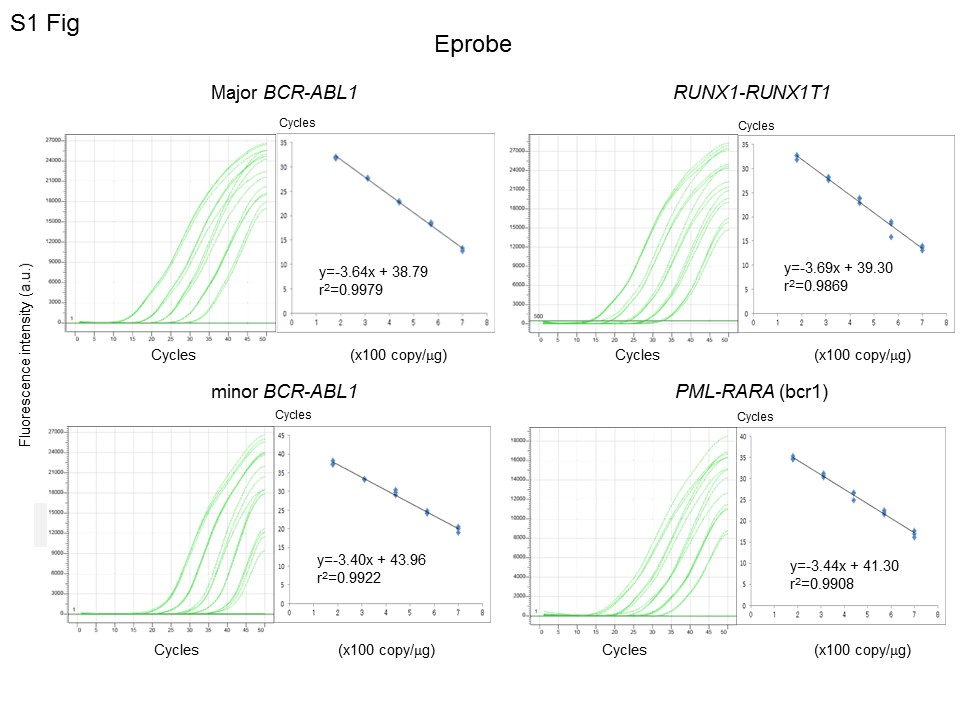

Supplement: S1 Fig — Eprobe mediated RT-qPCR experiments were performed with different concentrations of standard RNA. Amplification curve lines and PCR efficiency plots (threshold cycle [Ct] values plotted against the logarithm of standard RNA concentrations) are shown. RNA concentrations used for the Eprobe-based reaction were 62.5, 12.5 × 102, 2.5 × 10^4^, 5.0 × 10^5^, and 1.0 × 10^7^ copies/reaction. (JPG) [file pone.0202429.s004.JPG]

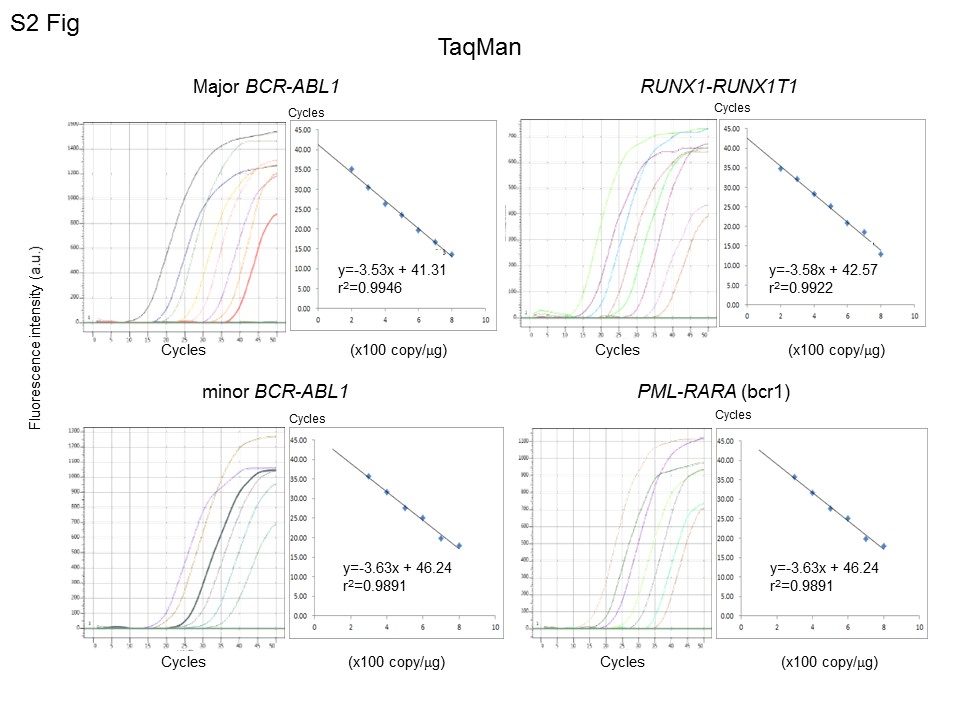

Supplement: S2 Fig — TaqMan RT-qPCR experiments were performed by the exact same methods described in S1 Fig. RNA concentrations used for the TaqMan probe-based reaction were 10, 102, 103, 104, 105, 106, 107, and 108 copies/reaction. (JPG) [file pone.0202429.s005.JPG]

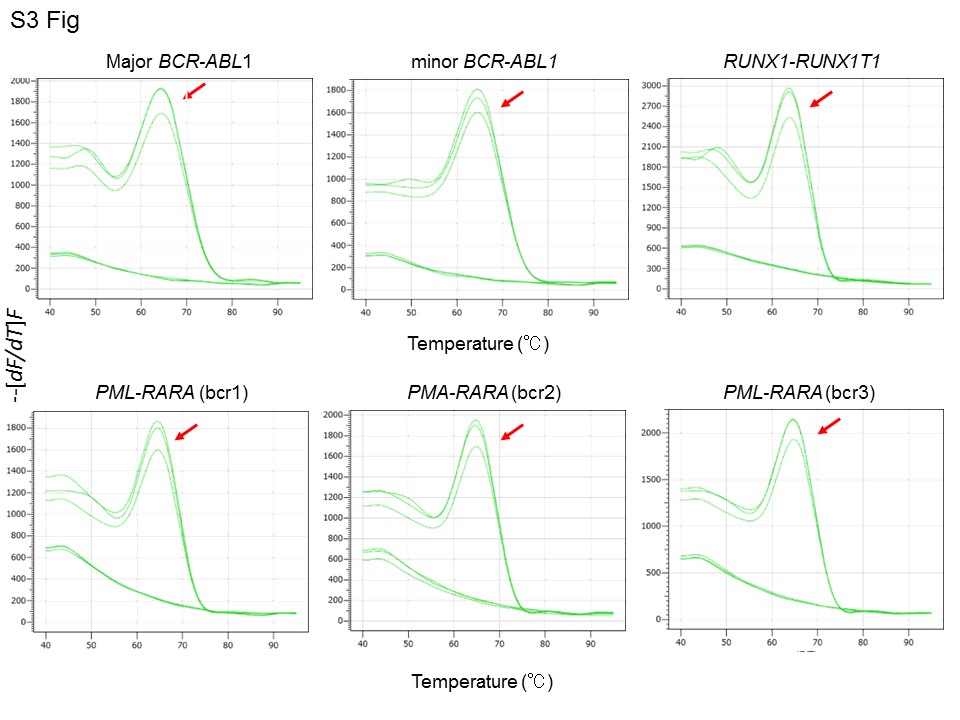

Supplement: S3 Fig — Results of melting curve analyses are shown as the first derivative curve by plotting fluorescence (F) variation (-[dF/dT]F) against temperature (T), which was derived from Eprobe mediated RT-qPCR products of the indicated leukemia-related fusion transcripts. Red arrow indicates the melting peak of each respective product. The theoretical Tm value of each targeted Eprobe is shown as follows: major and minor BCR-ABL1, 65.0°C; RUNX1-RUNX1T1, 62.5°C; PML-RARA (bcr1, bcr2, or bcr3), 64.7°C. Each theoretical Tm value was estimated using the ECHO/DNA Thermodynamics method19. (JPG) [file pone.0202429.s006.JPG]
